# Theological Scarecrows

**Published:** 1888-06

**Authors:** 


### THEOLOGICAL SCARECROWS.

We clip the subjoined item from the New York *Sun* :

The following words are reported to have been written by a missionary in Japan, concerning the effect of the preaching of the "orthodox Gospel" to the Japanese : "They grieve over the fate of their departed children, parents and relatives, and often show their grief by tears. They ask us if there is any hope, any way to free them by prayer from that eternal misery, and I am obliged to answer there is absolutely none. Their grief at this affects and torments them wonderfully ; they almost pine away with sorrow. They often ask if God cannot take their father out of hell, and why their punishment must never have an end. They do not cease to grieve, and I can hardly restrain my tears at seeing men so dear to my heart suffer such intense pain. Such thoughts have, I imagine, risen in the hearts of all missionary teachers of all churches."

"The knots that tangle human creeds" are not likely to be disentangled so long as the old and severe doctrines which make man a shuttlecock to be played between rival battledores, is taught as one of the theological belief in fields devoted to missionary enterprise. Indeed, from the foregoing item, it would appear that there is greater need of sending missionaries to missionaries than to the heathen.

